# Use of machine learning for behavioral distinction of autism and ADHD

**DOI:** 10.1038/tp.2015.221

**Published:** 2016-02-09

**Authors:** M Duda, R Ma, N Haber, D P Wall

**Affiliations:** 1 Department of Pediatrics, Division of Systems Medicine, Stanford University, Stanford, CA, USA; 2 Department of Biomedical Data Science, Stanford University, Stanford, CA, USA

## Abstract

Although autism spectrum disorder (ASD) and attention deficit hyperactivity disorder (ADHD) continue to rise in prevalence, together affecting >10% of today's pediatric population, the methods of diagnosis remain subjective, cumbersome and time intensive. With gaps upward of a year between initial suspicion and diagnosis, valuable time where treatments and behavioral interventions could be applied is lost as these disorders remain undetected. Methods to quickly and accurately assess risk for these, and other, developmental disorders are necessary to streamline the process of diagnosis and provide families access to much-needed therapies sooner. Using forward feature selection, as well as undersampling and 10-fold cross-validation, we trained and tested six machine learning models on complete 65-item Social Responsiveness Scale score sheets from 2925 individuals with either ASD (*n*=2775) or ADHD (*n*=150). We found that five of the 65 behaviors measured by this screening tool were sufficient to distinguish ASD from ADHD with high accuracy (area under the curve=0.965). These results support the hypotheses that (1) machine learning can be used to discern between autism and ADHD with high accuracy and (2) this distinction can be made using a small number of commonly measured behaviors. Our findings show promise for use as an electronically administered, caregiver-directed resource for preliminary risk evaluation and/or pre-clinical screening and triage that could help to speed the diagnosis of these disorders.

## Introduction

Autism spectrum disorder (ASD) and attention deficit hyperactivity disorder (ADHD) are among the most common childhood disorders, with the most recent prevalence estimates by the Centers for Disease Control at 1.5% and 9.5%, respectively.^1, 2^ ASD and ADHD have considerable behavioral overlaps, including impulsivity and trouble with social interactions.^3^ However, these behavioral overlaps can complicate differential diagnosis for clinicians. For example, a child with predominant ADHD might struggle with social interactions, but this may stem from inattention to the speaker or interrupting due to impulsivity, rather than a fundamental misunderstanding of social cues, which more strongly aligns to a child with autism.

This is not to say that autism and ADHD are mutually exclusive. In fact, the most recent Diagnostic and Statistical Manual (DSM-V)^4^ has recognized the frequency of co-occurrence of ASD and ADHD symptoms and has altered its diagnostic criteria, which previously precluded a dual-diagnosis under DSM-IV, to clinically formalize the autism-ADHD comorbidity.

Both ASD and ADHD are identified through extensive examination, including evaluation by a team of behavioral pediatricians and child psychologists as well as administration of a number of diagnostic assessments by certified professionals. These rigorous diagnostic examinations often last multiple hours, and the ever-increasing demand for these appointments far exceeds the maximum capacity for developmental pediatrics clinics across the country. This bottleneck translates to wait times of up to 13 months from initial concern to diagnosis,^5^ or even longer if from a minority group or lower socio-economic status,^6^ contributing to the average ages of diagnosis of 4.5 years for ASD and 7 years for ADHD, despite the fact that a majority of parents identify developmental concerns before 36 months.^1, 2^

In previous work, we applied machine learning to score sheets of two commonly used autism diagnostic tools, the Autism Diagnostic Observation Schedule^7^ and Autism Diagnostic Interview-Revised,^8^ to develop abbreviated classification algorithms that distinguish ASD from non-ASD with high accuracy.^9, 10, 11, 12^ Using analogous methods, we hypothesized that we could achieve similar success in differentiating ASD from ADHD. On surveying the data available in archived research records, the Social Responsiveness Scale (SRS)^13^ provided the largest sample size of ADHD subjects for our analysis. The SRS is a parent-directed questionnaire that consists of 65 questions, and is commonly used to assess severity of autistic traits. Here, we train and test six different machine learning algorithms on SRS data from 2925 subjects, implementing forward feature selection methods that are tailored to each algorithm to reduce the original set of 65 features to ⩽9 in every case with no statistical decrease in performance as measured against the best estimate clinical diagnosis provided by both the physician and parent. The resulting classifiers show promise for use as pre-clinical screening tools for the evaluation of ASD/ADHD risk.

## Materials and methods

### Data sample

We aggregated complete item-level score sheets of the SRS from the Simons Simplex Collection version 15, Boston Autism Consortium and Autism Genetic Resource Exchange for a total of 2775 subjects with autism and 150 subjects with ADHD (Table 1). The SRS consists of 65 parent-directed items concerning their child's behavior that are answered using a choice of 'Not True', 'Sometimes True', 'Often True', 'Always True', that corresponds to a numerical scale of 1–4, respectively. For our analyses, missing answers were coded as 0's, and we limited our data set to score sheets with <5 total missing answers.

Classification of ASD was provided as physician-reported clinical diagnosis and classification of ADHD was provided as parent-reported clinical diagnosis. The ASD subjects selected had no documented ADHD comorbidity, and the ADHD subjects selected did not have any documented diagnosis of autism. The ADHD group consisted mainly of siblings of the autism probands that reported a prior clinical diagnosis of ADHD. The ASD group was 83.9% male and 15.6% female (0.5% unknown). The ADHD group was 62% male and 37.3% female (0.7% unknown).

### Machine learning

We tested the performance of six machine learning algorithms on our data, using the 65 items in the SRS as features and the diagnosis of either ASD or ADHD as the prediction class. We initialized our machine learning pipeline by splitting the entire data set into 10 stratified folds, where each fold consisted of 10% of both the ASD data (*n*=2775) and ADHD data (*n*=150). We used these folds to perform 10-fold cross-validation, where each trial dedicated nine folds for training data and the remaining one fold for testing. For each cross-validation trial, feature ranking was performed on the nine folds in the training set. All 65 features were ranked based on mutual information, calculated from the minimal-redundancy-maximal-relevance (mRMR) criterion described by Peng *et al.*^14^ The mRMR score is a metric to quantify the contribution of a feature to a model, calculated by subtracting the predictive power of the feature (maximal-relevance) from its similarity to other features already in the model (minimal-redundancy).

After feature ranking on the training data was complete, we performed 10 random undersamplings on the ASD majority group in both the training and testing sets to achieve a ratio of 1.5:1 ASD to ADHD in each set. This subsampling technique was used to ameliorate the issue of significantly unbalanced classes, as well as to safeguard against any age or gender biases that may be inherent to the data. For each sampling, we implemented forward feature selection using the previously obtained feature rankings to train and test our six machine learning algorithms, recording the performance of each model with default parameters, as well as with parameter tuning for each choice of features. This process was repeated for each of the 10 cross-validation trials, resulting in an average area under the receiver operating characteristic (ROC) curve (AUC) for each model calculated over 100 trials. All machine learning analyses were performed in Python using the package Scikit-learn.^15^ The Support Vector Classification (SVC) model was implemented using the linear kernel, Categorical Lasso was implemented as Logistic Regression with l_1_ regularization and our other Logistic Regression model (referred to here as Logistic Regression) was implemented with l_2_ regularization.

## Results

For each trial in the 10-fold cross-validation, mutual information feature ranking was performed on the nine folds designated as the training set. In each of these 10 trials, the same 6 features were consistently identified as the top ranked features, and variation in feature order was not seen until rank 7 and beyond. The behaviors associated with the top six features can be found in Table 2. Supplementary Table 1 provides the average Mutual Information Rank across the 10 trials, as well as response breakdowns for both the ASD and ADHD groups and *X*^2^-values comparing these distributions, for all 65 SRS items.

**Figure 1:**
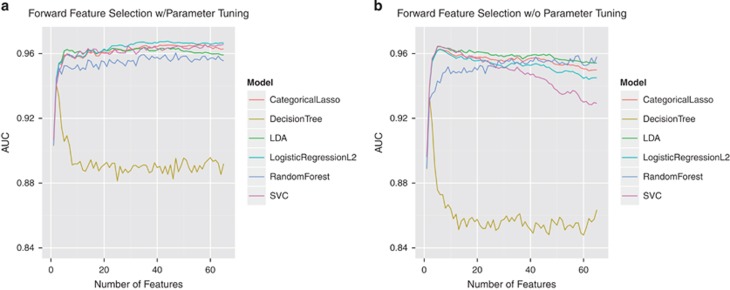
Forward feature selection results with and without parameter tuning. Using forward feature selection methods, we tested the performance of six machine learning algorithms at each feature addition, both with parameter tuning (**a**) and without (**b**). All of the six algorithms performed with peak AUC⩾0.93; five of these only required five features. AUC, area under the receiver operating characteristic curve; LDA, linear discriminant analysis; SVC, support vector classification.

Figure 1 illustrates the results of our forward feature selection process for each of our six algorithms, both with and without parameter tuning. To select the optimal number of features for each algorithm, we identified the point at which there was no considerable gain in accuracy, as measured by the AUC, when more features were added to the model. We found that five of the six algorithms performed close to the optimum using five features or less (Table 3). Most algorithms exhibited a gradual decline in performance as more features were added to the model (Figure 1a), which could often be corrected back to the baseline with heavier regularization, as evidenced by our parameter tuning results (Figure 1b). The heavier regularization prevented over-fitting on the training data, which was more likely to occur when less relevant (lower ranked) features were considered.

**Figure 2:**
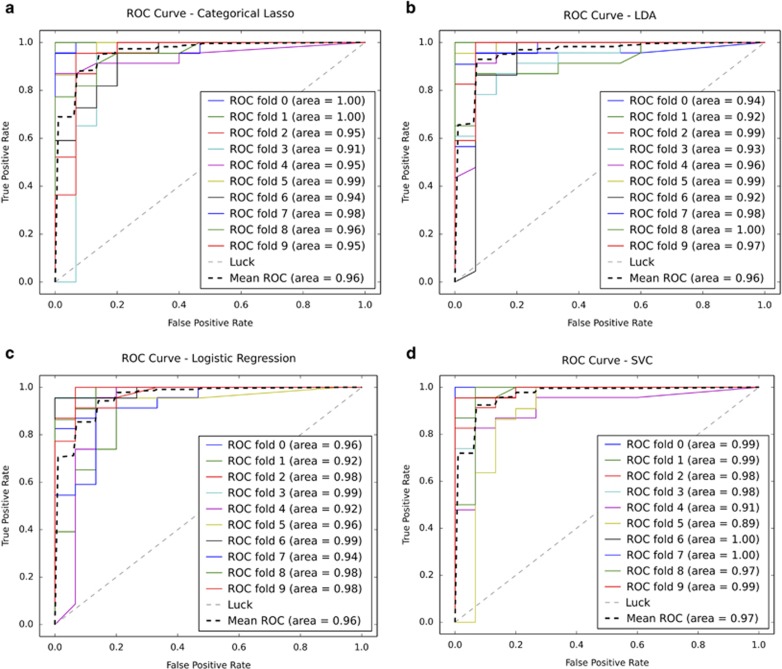
ROC curves of subsampled 10-fold cross-validation of top-performing algorithms. Using a random subsampling of the ASD set, we plotted the receiver operating characteristic (ROC) curves for each fold of our cross-validation, as well as the mean over all folds, for each of our four top-performing algorithms; Categorical Lasso (**a**), Logistic Regression (**b**), Support Vector Classification (**c**) and linear discriminant analysis (**d**). ASD, autism spectrum disorder.

**Figure 3:**
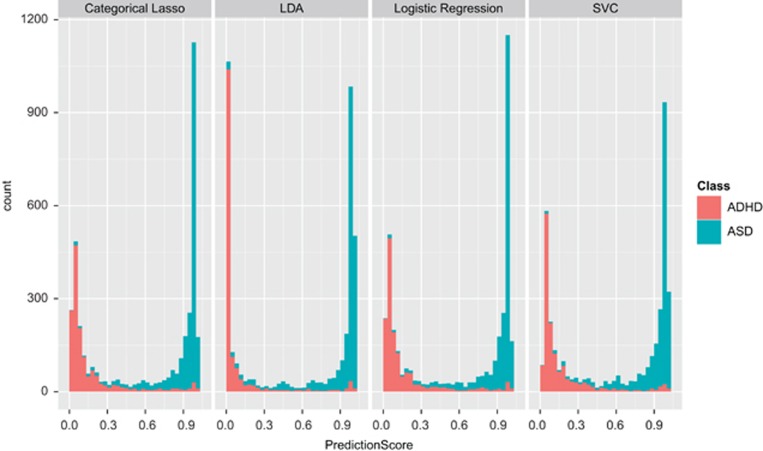
Distribution of probability scores of top-performing algorithms over 100 training/testing trials. These distributions show clear separation of the ASD and ADHD populations. The probabilistic nature of these models is beneficial, as prediction scores can be interpreted as confidence measures. ADHD, attention deficit hyperactivity disorder; ASD, autism spectrum disorder; LDA, linear discriminant analysis; SVC, support vector classification.

Four of our algorithms (SVC, LDA, Categorical Lasso and Logistic Regression) performed with comparable accuracy (0.962–0.965) utilizing the same five items, and represent the best models for this classification task. We compared ROC curves of these four classifiers applied to a subsample of our data using stratified 10-fold cross-validation (Figure 2), as well as the distributions of probability scores (Figure 3).

## Discussion

Behavioral diagnosis of both ASD and ADHD is a time-intensive process that can be complicated by the overlaps in symptomatology. Due to the high demand for the multi-hour clinical assessments necessary for diagnosis, many children are waitlisted for over a year, delaying their diagnosis and thereby delaying the start of behavioral and/or pharmaceutical interventions. Currently, there is no diagnostic instrument that can directly distinguish autism from ADHD, nor does there exist a screening tool that is expressly designed to distinguish risk between the two disorders with high accuracy. We have previously utilized machine learning techniques to successfully detect ASD from non-ASD^9, 10, 11, 12^ using a fraction of the behaviors that are traditionally measured, and others have performed similar analyses for detection of ADHD.^16^ Here, we sought to expand on these previous studies to test whether a limited set of behaviors derived from the SRS could be used to distinguish ASD from ADHD, thereby enabling increased specificity when confronted with clinically challenging cases. Using stratified 10-fold cross-validation, we implemented mutual information-based feature ranking and forward feature selection on six machine learning algorithms to develop classifiers that accurately separated these clinical populations using only a small number of features.

Machine learning automates part of the process of model building. Conventional methods use probabilistic assumptions to evaluate the validity of classification schemes that often amount to human-crafted thresholding of features. The various machine learning methods we employed allowed us to construct models optimized on the observed data, discovering new classification criteria. These methods have beneficial properties that are generally orthogonal to the typical probabilistic assumptions used in more conventional statistical tests (often relying on distribution assumptions of the population of interest). For instance, Logistic Regression with l_1_ regularization is a discriminative model that enforces sparsity, guarding against the use of irrelevant features. Support vector machines do not admit a simple Bayesian interpretation and build a model based on extreme examples—data points near where a decision boundary should be. These properties allow us to automatically build models with potentially very different and more effective properties than conventional analyses might yield.

Our results showed that the tree-based algorithms, Decision Tree and Random Forest, were not well-suited to the classification task at hand. In Figure 1, a notable drop in the performance of the Decision Tree model can be seen in both the default and parameter-tuned experiments. This is not entirely unexpected, considering the simplistic nature of this algorithm, and the limited safeguards it has against over-fitting. The Random Forest model exhibited sufficient classification performance but required nearly twice as many features as the other four models. This outcome is expected considering that the Random Forest algorithm relies on multiple Decision Trees to achieve a 'majority rules' classification model.

Conversely, we found that four of our six algorithms (SVC, LDA, Categorical Lasso and Logistic Regression) all performed with high accuracy (AUC>0.96) and utilized only five behaviors, representing a >92% reduction in the number of behaviors measured with the standard SRS. It is important to note that these five features were always chosen as the top-ranking features using the mRMR criterion in our 10 cross-validation trials, indicating that they are valuable for distinguishing between the two disorders. In fact, many of these features overlap with the behaviors that are measured in several of our previously created behavioral classifiers, including eye contact, reciprocal communication and peer play.^9, 10, 12^ These models proved to be optimal for our classification task, not only due to their low error rate, but also due to their probabilistic qualities. Since they provide a probability score in addition to a classification decision, these models provide the ability to interpret their output in terms of confidence in the classification and subsequently in terms of risk level for either disorder (Figure 3).

Our results suggest the possibilty that one of these classifiers, used singly or in conjunction with our previously created behavioral classifiers explicitly focused on differentiating autism from a more encompassing non-autism category, could act as a useful pre-clinical screening and triage tool to assess risk of ADHD and autism. Though the SRS is indicated for use in children aged 4–18 years, the behaviors that are measured by this tool are highly adaptable to younger ages, as is evidenced by the SRS-Preschool screening instrument, which implements a relatively small number of wording changes to make the 65 questions suitable for children as young as 3 years. This suggests the possibility that our classifier could be similarly adapted to this age group, and perhaps even younger. Given that the average age of diagnosis is currently 4.5 years for ASD and 7 years for ADHD and that roughly 90% of parents identify developmental concerns before 36 months,^1, 2^ there is strong impetus to focus energy on developing faster and mobilized systems for risk detection. Our results support the hypothesis that detection of ASD/ADHD risk earlier than these national averages is possible. However, further testing and validation must be done to confirm the accuracy in independent replicate samples.

Furthermore, the caregiver-directed nature of our classifier coupled with the brevity of the question set, stresses a novel opportunity to create a mobile screening platform that could have broad access in terms of both geographic location and socio-economic status. By delivering this screening system via a mobile app, parents with developmental concerns about their child could receive an accurate assessment of their child's risk for ASD/ADHD more quickly than what is possible today. Moreover, if adapted by developmental medicine clinics, such a mobilized screening platform could prove useful for patient triage, enabling higher throughput and improved waitlist management.

Further evaluation of this classification system is needed, including validation on a larger population of ADHD subjects, as well as broadening the classifiers to handle multi-label classification of individuals that have both ASD and ADHD. However, these preliminary results support the possibility that machine learning classifiers can be used as a short, comprehensible, caregiver-directed screening measure that, on a mobile platform, could provide a quick and streamlined assessment of risk.

### Limitations

In this analysis of archival data, we were limited by the content of the data sets available. Given that these data stemmed from primarily autism-based collections, there was a large imbalance in favor of the ASD class (Table 1). The 150 ADHD subjects we were able to identify were unaffected siblings of ASD probands that had reported a previous diagnosis of ADHD. We were able to circumvent this class imbalance through stratified 10-fold cross-validation and repeated random undersampling, allowing the classifiers to be trained on 90% of the data and tested on the remaining 10% of the data independently. Our constrained sample size prevented us from devoting a portion of our data exclusively to validation; however, our models were trained and tested on 100 unique combinations of ASD and ADHD subjects (10 cross-validation trials × 10 samplings per trial). While this methodology allowed us to utilize as much of the data as possible and provides a robust estimate of the performance of our algorithms on new data, a subsequent validation study with a larger ADHD cohort is needed, and currently underway, to further assess the performance of these classifiers.

## Figures and Tables

**Table 1:** Sample description

	*SSC*		*AGRE*		*AC*	
	*ASD*	*ADHD*	*ASD*	*ADHD*	*ASD*	*ADHD*
Sample size	2394	133	400	17	4	0
						
*Age*						
Q1	70.75	109.5	99.69	103.56	58	0
Median	97	134.5	129.36	133.4	64.5	0
Q2	132	163.5	163.08	156.36	71	0
IQR	61.25	54	63.69	52.8	13	0

Abbreviations: AC, Boston Autism Consortium; ADHD, attention deficit hyperactivity disorder; AGRE, autism genetic resource exchange; ASD, autism spectrum disorder; IQR, inter-quartile range; Q1, first quartile; Q3, third quartile; SSC, Simons Simplex Collection.

All ages are in months.

**Table 2:** Feature rankings

*Rank*	*SRS question*
1	35. Trouble with the flow of normal conversation
2	24. Difficulty with changes in routine
3	22. Appropriate play with peers
4	37. Difficulty relating to peers
5	16. Atypical or inconsistent eye contact
6	29. Regarded by other children as 'odd'

Abbreviation: SRS, Social Responsiveness Scale.

Top-ranking behavioral features as identified by the minimal-redundancy-maximal-relevance (mRMR) criterion.

**Table 3:** Accuracy of machine learning algorithms

*Algorithm*	*AUC*	*Features used*
Decision tree	0.933	2/65
Random forest	0.952	9/65
Support Vector Classification	0.965	5/65
Logistic Regression	0.962	5/65
Categorical lasso	0.962	5/65
Linear discriminant analysis	0.964	5/65

Using mutual information feature selection methods. Categorical Lasso was implemented as a Logistic Regression model with l_1_ regularization and our Logistic Regression model utilized l_2_ regularization. Support Vector Classification was applied using the linear kernel.
